# Metagenomic 16S rRNA analysis and predictive functional profiling revealed intrinsic organohalides respiration and bioremediation potential in mangrove sediment

**DOI:** 10.1186/s12866-024-03291-8

**Published:** 2024-05-22

**Authors:** Sultan M. Alsharif, Mohamed Ismaeil, Ali M. Saeed, Wael S. El-Sayed

**Affiliations:** 1 Department of Biology, College of Science, Taibah University, Al-Madinah, Kingdom of Saudi Arabia; 2 Microbiology Department, Faculty of Science, Ain Shams University, Cairo, Egypt

**Keywords:** Metagenomic analysis, 16S rRNA, Mangrove sediment, Organohalides bioremediation

## Abstract

**Background:**

Mangrove sediment microbes are increasingly attracting scientific attention due to their demonstrated capacity for diverse bioremediation activities, encompassing a wide range of environmental contaminants.

**Materials and methods:**

The microbial communities of five *Avicennia marina* mangrove sediment samples collected from Al Rayyis White Head, Red Sea (KSA), were characterized using Illumina amplicon sequencing of the 16S rRNA genes.

**Results:**

Our study investigated the microbial composition and potential for organohalide bioremediation in five mangrove sediments from the Red Sea. While *Proteobacteria* dominated four microbiomes, *Bacteroidetes* dominated the fifth. Given the environmental concerns surrounding organohalides, their bioremediation is crucial. Encouragingly, we identified phylogenetically diverse organohalide-respiring bacteria (OHRB) across all samples, including *Dehalogenimonas*, *Dehalococcoides, Anaeromyxobacter, Desulfuromonas, Geobacter*, *Desulfomonile*, *Desulfovibrio*, *Shewanella* and *Desulfitobacterium*. These bacteria are known for their ability to dechlorinate organohalides through reductive dehalogenation. PICRUSt analysis further supported this potential, predicting the presence of functional biomarkers for organohalide respiration (OHR), including reductive dehalogenases targeting tetrachloroethene (PCE) and 3-chloro-4-hydroxyphenylacetate in most sediments. Enrichment cultures studies confirmed this prediction, demonstrating PCE dechlorination by the resident microbial community. PICRUSt also revealed a dominance of anaerobic metabolic processes, suggesting the microbiome’s adaptation to the oxygen-limited environment of the sediments.

**Conclusion:**

This study provided insights into the bacterial community composition of five mangrove sediments from the Red Sea. Notably, diverse OHRB were detected across all samples, which possess the metabolic potential for organohalide bioremediation through reductive dehalogenation pathways. Furthermore, PICRUSt analysis predicted the presence of functional biomarkers for OHR in most sediments, suggesting potential intrinsic OHR activity by the enclosed microbial community.

## Introduction

Distributed along tropical and subtropical coastlines at the land-sea interface, mangrove ecosystems occupy a unique environmental niche [1, 2]. Notably, mangrove forests play pivotal roles in ecosystem function by filtering and reducing both dissolved and particulate nutrients. Additionally, they serve as crucial reservoirs for carbon, nitrogen, and phosphorus, even sequestering heavy metals from adjacent terrestrial environments [3]. These unique conditions render mangrove sediments ideal habitats for diverse microbial communities to thrive.

Mangrove-associated microbial communities exert significant influence on biogeochemical cycling within these ecosystems, facilitating the transformation of carbon, sulfur, nitrogen, and phosphorus through diverse metabolic pathways [1]. Furthermore, the complex and phylogenetically diverse nature of mangrove microbiota underpins their critical roles in maintaining ecosystem productivity and facilitating post-disturbance recovery processes within these vital habitats [4].

Mangrove sediments, characterized by their high nutrient content, serve as a rich habitat fostering the proliferation of diverse bacterial communities with potent metabolic and bioremediation capabilities [5, 6]. Mangrove ecosystems act as biogeochemical sinks for various xenobiotic pollutants, including polychlorinated biphenyls (PCBs), heavy metals, and polycyclic aromatic hydrocarbons (PAHs) [7–9]. This widespread accumulation suggests the presence of a robust microbial community harboring diverse bioremediation capabilities.

Studies have identified a wide range of phylogenetically diverse bacterial genera residing within this environment, including *Pseudomonas, Marinobacter, Alcanivorax, Microbulbifer, Sphingomonas, Micrococcus, Cellulomonas, Dietzia and Gordonia*. Notably, these bacteria have exhibited the ability to degrade harmful hydrocarbons, such as polycyclic aromatic hydrocarbons (PAHs) [10, 11]. Furthermore, research suggests that mangrove sediments possess significant potential for bioremediation strategies, demonstrating efficacy in the decontamination of oil spills [12]. Metagenomic analysis of oil-contaminated mangrove sediments identified *Geobacter, Rhodopseudomonas, and Pseudomonas*, *Shewanella, Anaeromyxobacter, Rhodopirellula, and Thioalkalivibrio* as the dominant bacterial genera [13].

Chloroethene contaminants, such as tetrachloroethene (PCE), trichloroethene (TCE), and dichloroethane (DCE), are prevalent in groundwater due to their extensive use in industry as solvents and degreasers [14]. Their persistence and potential to cause cancer necessitate their classification as priority pollutants, requiring monitoring across diverse environmental settings. Microbial reductive dechlorination, a process extensively documented in anaerobic groundwater systems, serves as the primary mechanism for degrading PCE and TCE, potentially mitigating their environmental impact [15, 16]. This process relies on organohalide-respiring bacteria (OHRB), which utilize organohalide compounds as their final electron acceptors for energy production [17]. The environmental significance of OHRB-mediated dechlorination, particularly in sedimentary systems, has attracted significant research interest due to its potential for natural bioremediation [18, 19]. This biogeochemical phenomenon exhibits near-ubiquity in anaerobic aquifers [15, 16, 20–22] and extends to a diverse array of sedimentary environments [23–26], highlighting its widespread ecological importance.

This study investigates the potential for microbial-mediated dechlorination of organohalides within the mangrove ecosystem. Illumina amplicon sequencing of the 16S rRNA gene will be employed to characterize the microbiome composition of mangrove sediment samples. Given the established role of organohalide respiration (OHR) in anaerobic bioremediation of toxic organohalides, the study further aims to detect the presence of phylogenetically diverse OHRB. Additionally, efforts will be made to predict functional OHR biomarkers and directly assess in situ organohalide dehalogenation activity within an enrichment culture simulating the mangrove sediment environment.

## Materials and methods

### Sampling site and samples collection

**Fig. 1:**
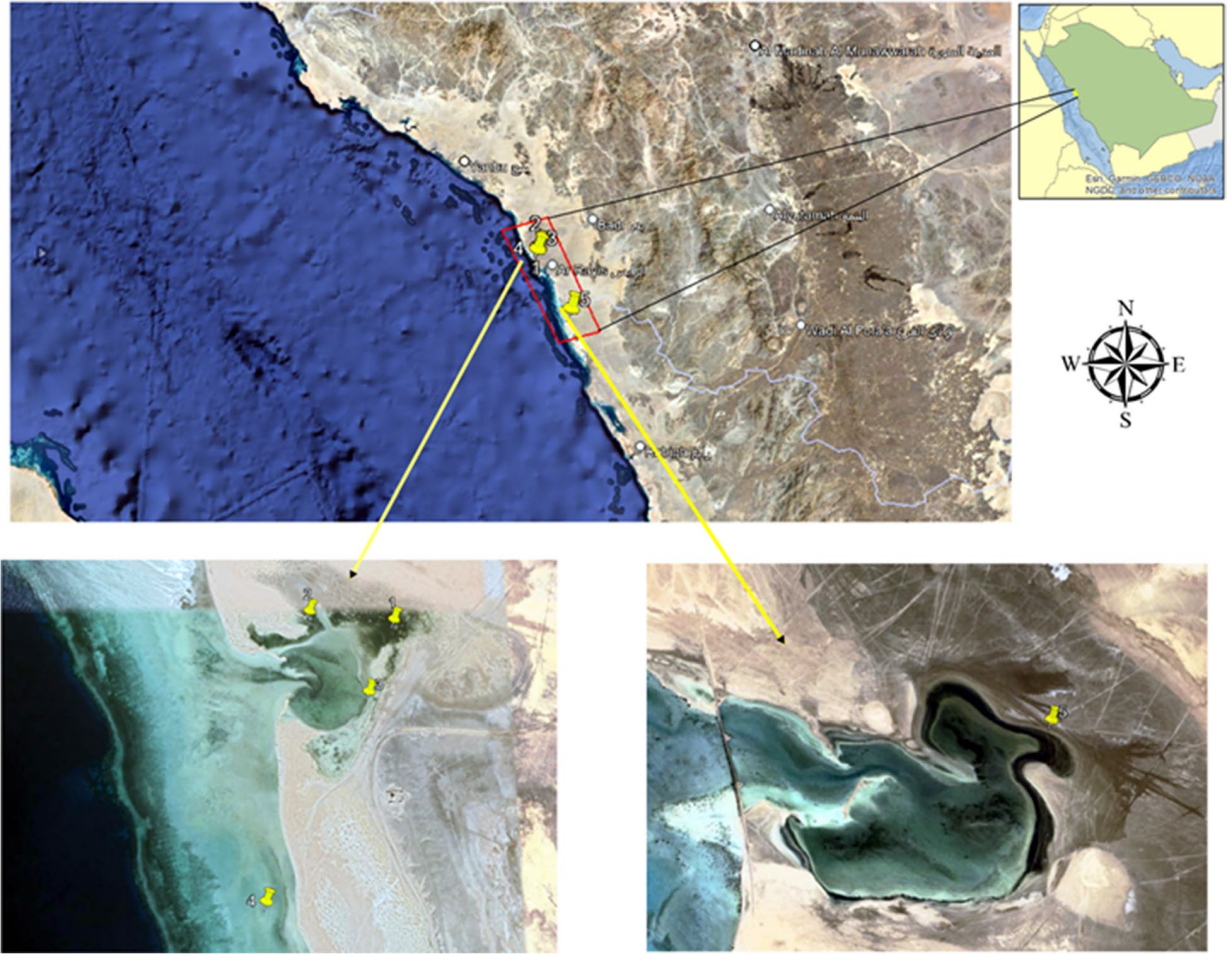
Geographical map showing sampling sites for the five samples addressed by this study

Five mangrove sediment samples designated MS1-MS5 were collected from the root zone of *Avicennia marina* stands (MS1, 23°35’57.2"N 38°32’39.5"E; MS2, 23°35’58.3"N 38°32’28.3"E; MS3, 23°35’47.9"N 38°32’36.1"E; MS4, 23°35’23.3"N 38°32’23.4"E; MS5, 23°19’59.7"N 38°41’52.3"E) along the shore of Al Rayes city, Red Sea, KSA (Fig. 1). *Avicennia marina* is the dominant mangrove species in the marine environment of the Al Rayes White Head region and along the shore of Al Rayes city. They typically range in height from 1 to 4 m and possess light gray bark with stiff flakes and thick, glossy, and bright green leaves on the upper side and gray or silvery white with small hairs on the lower side. Mangrove sediment samples were collected from various locations to a depth of approximately 0.15 m using pre-sterilized polyethylene bags. The collected samples were then placed in sterile containers, sealed tightly, and transported to the laboratory for further microbiological analyses.

### Genomic DNA extraction, PCR amplification and Illumina amplicon sequencing

Total genomic DNA was directly extracted from approximately 1.0 g of mangrove sediment using an UltraClean Soil DNA purification kit (Mo Bio Laboratories, Solana Beach, CA, USA) according to the manufacturer’s protocol. Subsequently, PCR amplification targeted the bacterial hypervariable region V3-V4 of the 16S rRNA gene using the universal primer pair 341 F (CCTACGGGNGGCWGCAG) and 805R (GACTACHVGGGTATCTAATCC) [27, 28]. The reaction followed the Illumina 16S rRNA Metagenomic Sequencing Library protocol (www.illumina.com) and was performed at Macrogen sequencing facility (Seoul, South Korea). The amplification program included an initial denaturation step at 95 °C for 3 min, followed by 25 cycles of denaturation at 95 °C for 30 s, annealing at 55 °C for 30 s, and extension at 72 °C for 30 s. A final extension cycle at 72 °C for 5 min concluded the program. The resulting PCR amplicons (~ 450 bp) were sequenced using the Illumina MiSeq platform (2 × 300 bp paired-end reads) at Macrogen (Seoul, South Korea).

### Bioinformatics and diversity analysis

Following 16S rRNA gene amplicon sequencing, taxonomic assignment and downstream statistical analyses were performed on raw reads using the MG-RAST platform [29]. Low-quality sequences were trimmed from FASTQ files uploaded to MG-RAST using SolexaQA [30]. Potential human sequences were subsequently removed with Bowtie [31], leaving only high-quality reads for further analysis. Sequences were annotated against the SILVA SSU Ref database [32] with a minimum identity of 60%, maximum alignment length of 15 bp, and an e-value of 1e-5. Downstream bioinformatic and statistical analyses were conducted using the 16S rRNA gene Microbiome Taxonomic Profiling (MTP) pipeline (https://www.ezbiocloud.net/contents/16smtp) on an EzBioCloud server [33]. Paired-end reads were merged after uploading using VSEARCH version 2.13.4 [34] and filtered for low-quality (< Q25), chimeric, and non-target amplicons. Operational taxonomic units (OTUs) were then clustered at 97% sequence similarity using UCLUST [35] and CDHIT [36] softwares integrated within the EzBioCloud server. OTU picking and annotation were performed against the server’s PKSSU4.0 database [33]. Alpha-diversity indices, including Good’s coverage, rarefaction, observed OTUs, Simpson, Shannon, and Abundance-based Coverage Estimator (ACE), were then calculated. Beta diversity was determined using the UniFrac distance metric and principal coordinate analysis (PCoA). PCoA analysis was also calculated by the EzBioCloud server, conducted at the genus level and visualized using XLSTAT (Addinsoft, New York, USA) software. A correlation analysis was done using the online free server, Science and research online plot (https://www.bioinformatics.com.cn/en). The *p* significance level was set at 0.05. A Venn diagram depicting unique and shared OTUs across samples was generated using the InteractiVenn online tool (http://www.interactivenn.net/).

### Predictive functional profiling and phylogenetic analysis

Predictive functional profiling was performed using the Phylogenetic Investigation of Communities by Reconstruction of Unobserved States (PICRUSt) tool embedded in EzBioCloud [37] in conjunction with Kyoto Encyclopedia of Genes and Genomes (KEGG) databases [38]. The PICRUSt tool uses the Kruskal-Wallis H test to identify significantly.

different functional profiles on a cutoff value of *P* < 0.05 [39]. Heatmaps visualizing functional biomarker distribution were generated with the SRplot online tool (https://www.bioinformatics.com.cn/en). Finally, a phylogenetic tree for identified genera was constructed using the neighbor-joining method in MEGA X software [40] and visualized with the Interactive Tree of Life (ITOL), (https://itol.embl.de).

### Establishment of mangrove sediment enrichment cultures

Mangrove sediment enrichment cultures were established in 100 mL sterile Duran bottles equipped with leak-proof screw caps. Each bottle contained 20% (w/v) sediment prepared in a basal mineral medium composed of (per liter): K_2_HPO_4_, 4.36 g; NaH_2_PO_4_, 3.45 g; (NH_4_)_2_SO_4_, 1.26 g; MgSO_4_.6H_2_O, 0.91 g; trace salt solution, 1 ml. Trace salt solution contained (per 100 ml): CaCl_2_.2H_2_O, 4.77 g; FeSO_4_.7H_2_O, 0.37 g; CoCl_2_.6H_2_O, 0.37 g; MnCl_2_, 0.1 g; Na_2_MoO_4_.2H_2_O, 0.02 g. Cultures were spiked with PCE (Sigma-Aldrich, Deisenhofen, Germany) as an electron acceptor to a final concentration of 100 µM. An unspiked culture served as a control. Established enrichment cultures were then injected with hydrogen gas as an electron donor and incubated at 30 °C for one month before further analysis [41].

### Gas chromatography/FID analysis

Chlorinated ethenes were analyzed by using a high-resolution gas chromatograph (GC) equipped with a flame ionization detector (FID) (Thermo Scientific Trace 1300 series). Ten µL samples were withdrawn from the headspace of established enrichment cultures and directly injected into the GC using a split/splitless injector. Separation of chlorinated ethenes was achieved on a TG-5MS fused silica capillary column (Restek, USA; 30 m, 0.25 mm ID, 0.25 μm film thickness) using the following temperature program: initial oven temperature of 40 °C held for 3 min, followed by a ramp to 80 °C at 8 °C/min, then another ramp to 190 °C at 44 °C/min, with a final hold at 190 °C for 5 min. Injector and detector temperatures were maintained at 260 °C and 300 °C, respectively. Splitless injection mode was employed with helium as the carrier gas [42].

## Results

### Characterization of mangrove sediment bacterial community

**Fig. 2:**
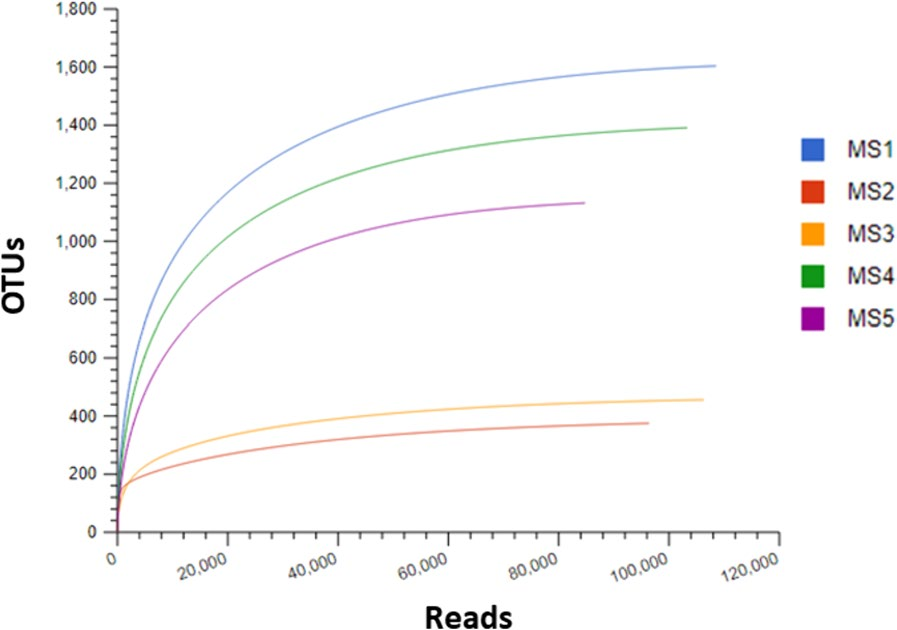
Rarefaction curves for the five mangrove-microbiomes identified in this study

Five mangrove sediment samples were examined to unveil their bacterial community structure (microbiome) using Illumina MiSeq sequencing of the 16S rRNA gene. The valid reads values were ranged from 99.16 to 99.16% (Table 1). The sequencing effort yielded a total of 47,419 to 92,882 reads per sample, encompassing 301 to 4467 operational taxonomic units (OTUs) (Table 1). Variations in OTUs composition across mangrove sediments might be associated with ecological factors influencing community structure along Al Rayyis White Head (Red Sea, KSA). Sampling sites MS1, MS4, and MS5 exhibited well-developed mangrove trees, forming dense vegetation, while sites MS2 and MS3 were characterized by patches of dwarf mangroves with limited stands of *Avicennia marina*. These contrasting plant communities could contribute to the observed differences in microbial diversity and community composition. High Good’s coverage scores (> 99%) (Table 1) indicated comprehensive capture of the resident bacterial communities, further supported by plateauing rarefaction curves (Fig. 2), demonstrating adequate sequencing depth for accurate diversity assessment.

**Table 1:** Alpha diversity indices (valid reads, valid reads percentage, OTUs obtained and Good’s coverage of library (%)) identified in our microbiomes

Sample name	Valid reads	Valid reads percentage	OTUs	Good’s coverage of library (%)
MS1	56,029	56.0%	4467	99.33
MS2	82,916	92.3%	301	99.98
MS3	92,878	92.9%	731	99.94
MS4	61,222	63.4%	3359	99.46
MS5	47,419	59.8%	2425	99.16

### Diversity analysis of bacterial communities

**Fig. 3:**
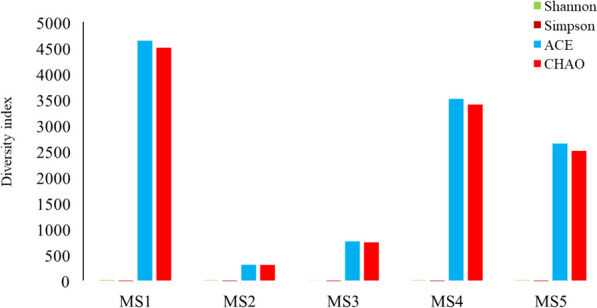
Diversity indices for the five mangrove microbiomes identified in this study

**Fig. 4:**
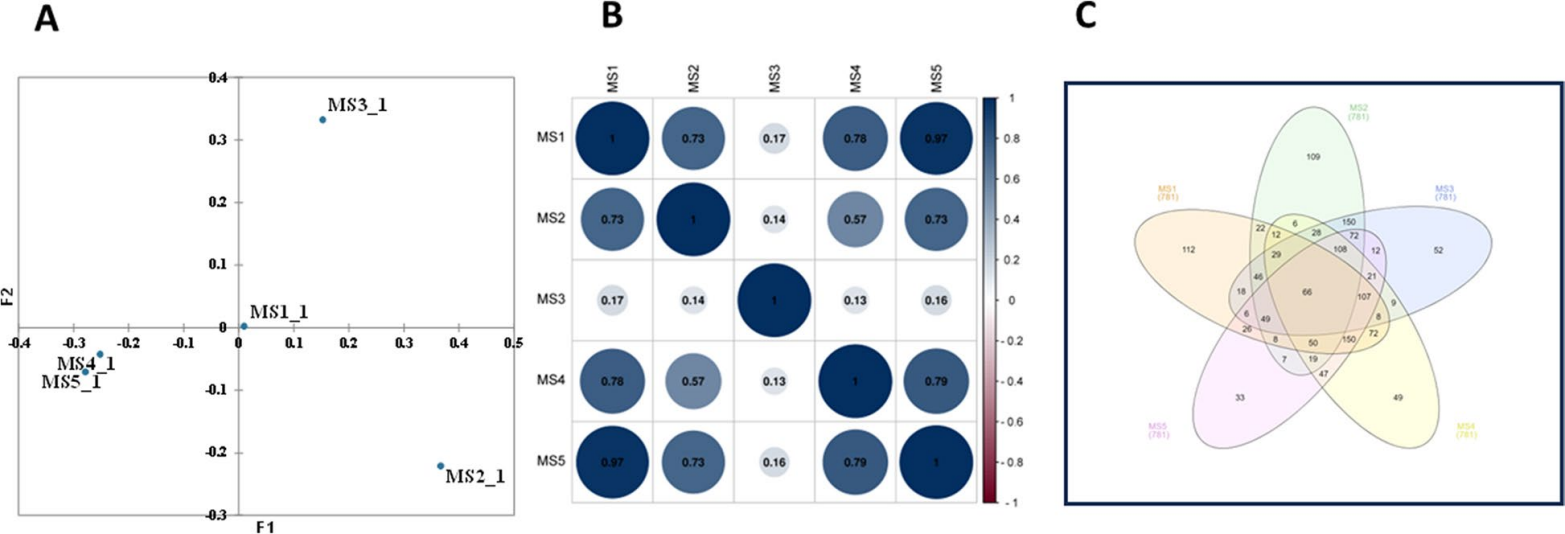
Similarity (or dissimilarity) between the five microbiomes where (**A**), PCoA plot showing clustering of the five microbiomes obtained in this study, the plot was performed based on weighted UniFrac distances. (**B**), Venn diagram showing the shared and the unique OTUs for the microbiomes obtained in this study

Shannon, ACE, and Chao1 indices revealed significantly higher alpha diversity in MS1, MS4, and MS5 compared to MS2 and MS3, suggesting greater richness and evenness within these microbial communities (Fig. 3). Conversely, the Simpson index indicated lower dominance in MS1, MS4, and MS5, further supporting diverse and less skewed taxonomic distributions. PCoA analysis (Fig. 4A) visualized distinct beta diversity patterns across sampling sites. Samples clustered into four distinct groups: MS1, MS2, MS3, and a combined group for MS4 and MS5, highlighting substantial compositional dissimilarities between sites. A correlation analysis (Fig. 4B) showed a positive correlation between all samples. The highest positive correlation value was identified between MS1 and MS samples, while the lowest one was found between MS3 and MS4 samples. Further, the Venn diagram (Fig. 4C) identified 66 OTUs shared across all microbiomes, while 112, 109, 52, 49, and 33 OTUs were unique to MS1, MS2, MS3, MS4, and MS5, respectively, emphasizing site-specific differences in bacterial taxa.

### Comparative analysis of mangrove microbiome

**Fig. 5:**
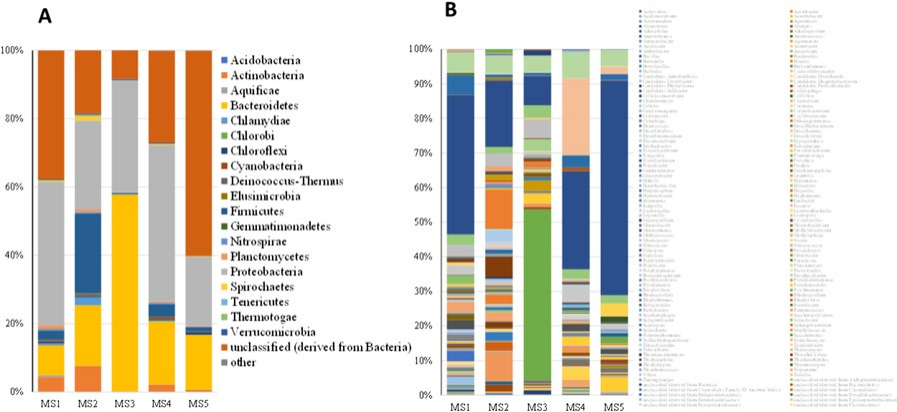
Bacterial community composition associated with the five mangrove sediments at phylum level (**A**) and genus level (**B**). Phyla and genera that took up < 0.01% of our microbiomes were identified together as other in the figure

Taxonomic classification of sequencing reads from the five mangrove microbiomes was performed using the SILVA reference database and the MG-RAST pipeline. At the phylum level (Fig. 5A), the *Proteobacteria* was the dominating phylum 3 of our 5 samples, accounting for 42.5%, 26% and 45.8% of the identified taxa in MS1, MS2 and MS4 samples, respectively. Unclassified bacterial sequences (18.6–60%) and *Bacteroidetes* (8.8–57.8%), *Actinobacteria* (0.2–4.2%) and *Firmicutes* (0.4–23.3%) followed in abundance across most samples. Notably, MS3 exhibited a unique phylum composition, with *Bacteroidetes* (57.6%) exceeding *Proteobacteria* (33%) in abundance. Interestingly, MS5 diverged by harbouring the highest proportion of unclassified bacterial sequences (60%). At the genus level, (Fig. 5B) the majority of sequences were assigned to unclassified derived from bacteria (37.6%), unclassified derived from *Gammaproteobacteria* (5.3%) and unclassified derived from *Deltaproteobacteria* (5.2%) in MS1 sample, unclassified derived from bacteria (18.5%), *Pseudoalteromonas* (11%) and *Aquimarina* (8.2%) in MS2 sample, *Flammeovirga* (48.4%), unclassified derived from bacteria (8.4%) and *Vibrio* (5.4%) in MS3 sample, unclassified derived from bacteria (26.9%), unclassified derived from *Epsilonproteobacteria* (21%) and unclassified derived from *Gammaproteobacteria* (7.1%) in MS4 sample and unclassified derived from bacteria (60%), *Gammaproteobacteria* (4.5%) and Arenibacter (4%) in MS5 sample. These observations highlight distinct phylum and genus levels profiles across the mangrove sediment microbiomes, suggesting potential functional and ecological variations.

### Distribution and abundance of OHRB

**Fig. 6:**
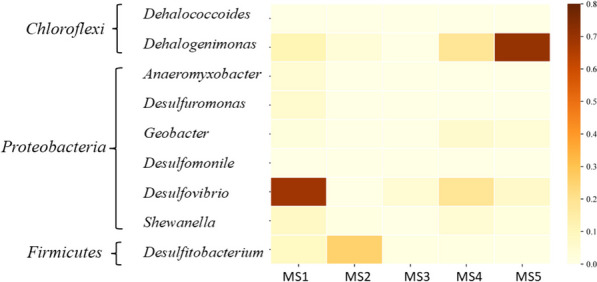
Heatmap plot showing the OHRB identified in the five microbiomes obtained in this study

**Fig. 7:**
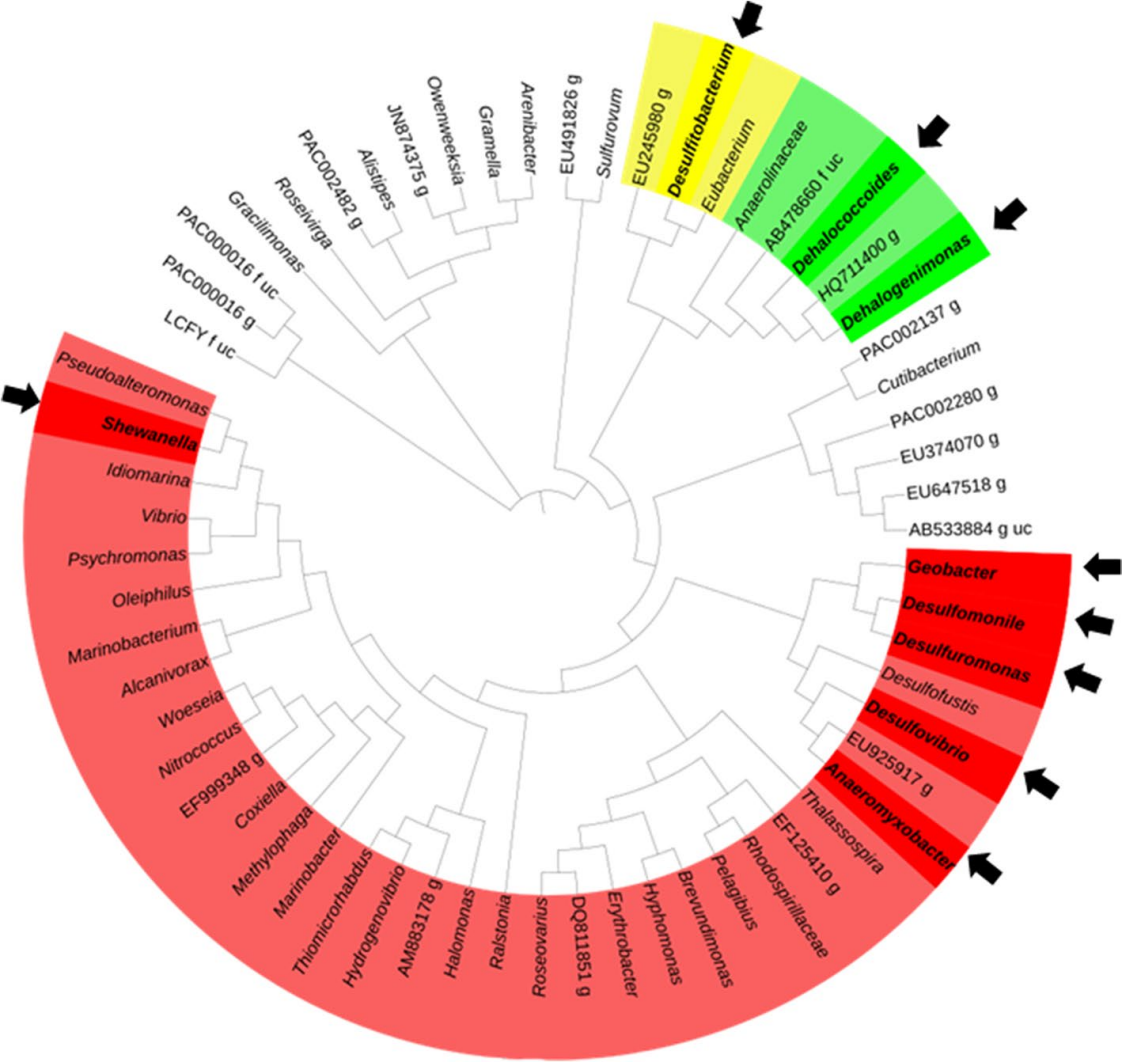
16s rRNA-based circular phylogenetic tree showing all genera obtained in this study. Genera belonging to phyla *Firmicutes*, *Chloroflexi* and *Proteobacteria*, identified so far to harbor OHRB, were highlighted in the yellow, green, and red color, respectively. black arrows refer to genera of OHRB identified in this study. OHRB genera were shown also in bold font

Known organohalide-respiring bacteria (OHRB) predominantly reside within the phyla *Chloroflexi*, *Proteobacteria*, and *Firmicutes* [43]. In this study, *Chloroflexi* exhibited a small relative abundance (< 1%) across all samples (Fig. 5A), Conversely, *Proteobacteria* and *Firmicutes* displayed markedly higher relative abundance values ranged from 20 to 45.8% and 0.8–23.3%, respectively (Fig. 5A). OHRB genera can be categorized as either obligate or versatile based on their sole reliance on OHR for energy acquisition [44]. Figure 6 depicts obligate OHRB genera *Dehalococcoides* and *Dehalogenimonas* belonging to *Chloroflexi*. Versatile OHRB genera, encompassing *Anaeromyxobacter*, *Desulfuromonas*, *Geobacter*, *Desulfomonile*, *Desulfovibrio*, and *Shewanella* from *Proteobacteria*, and *Desulfitobacterium* from *Firmicutes*, have been detected in mangrove sediments (Fig. 7).

### Potential organohalide respiration and diverse metabolic activities

**Fig. 8:**
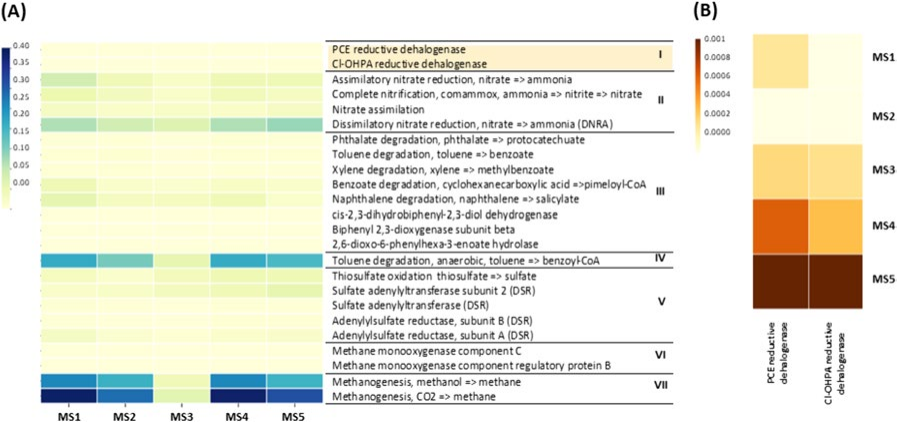
PICRUSt-constructed heatmap plot showing predictive functional biomarkers where (**A**) represents the overall major metabolic biomarkers identified as I, OHR; II, Nitrogen cycle; III, Aerobic hydrocarbon degradation; IV, Anaerobic hydrocarbon degradation; V, Sulfur cycle; VI, Methane oxidation and VII, Methanogenesis, while (**B**) represents identified biomarkers for OHR. The heatmap was constructed using EZ-biocloud server based on taxonomic 16S rRNA gene sequences

PICRUSt functional predictions identified two key OHR biomarkers: PCE reductive dehalogenase (KEGG KO K21647) and 3-chloro-4-hydroxyphenylacetate (Cl-OHPA) reductive dehalogenase (KEGG KO K21566) (Fig. 8A, B). These findings, coupled with the taxonomic presence of known OHRB genera (Figs. 6 and 7), suggest potential OHR activity within the sediment samples. Beyond OHR, PICRUSt revealed functional biomarkers for diverse metabolic activities across the samples (Fig. 8A). Notably, biomarkers associated with anaerobic processes, such as dissimilatory nitrate reduction to ammonia (DNRA), anaerobic hydrocarbon degradation, and methanogenesis, displayed high relative abundances. These findings suggest a rich and potentially complex metabolic landscape within the studied mangrove sediments.

### Confirmation of dechlorination activity and bioremediation potential

**Fig. 9:**
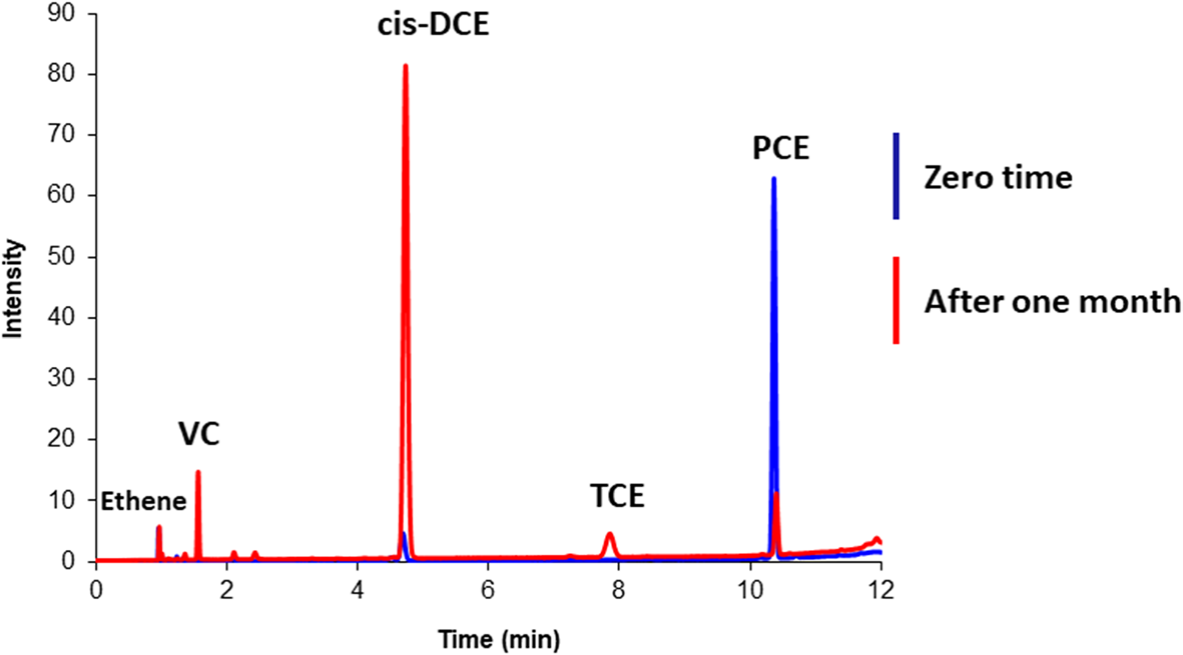
PCE dechlorination in mangrove sediment-amended enriched culture

Dechlorination activity within the studied mangrove sediment was confirmed through gas chromatography-flame ionization detection (GC-FID) analysis (Fig. 9). The chromatogram demonstrated the successful dechlorination of PCE to its daughter products, including trichloroethene (TCE), dichloroethene (DCE), vinyl chloride (VC), and ultimately, ethene. This confirms the presence and activity of dechlorinating microorganisms in the sediment. The bioremediation potential of the collected mangrove sediments was further evaluated through an enrichment culture study using PCE as a representative organohalide contaminant. This choice was based on its frequent detection as a groundwater pollutant and its known carcinogenicity. A comparative analysis of our findings with previously reported PCE dechlorination data (Table 2) suggests that various OHRB may be involved in PCE dechlorination to TCE and DCE, while *Dehalococcoides*, the only identified OHRB with documented DCE-to-ethene dechlorination activity, likely drives the final step.

**Table 2:** Potential OHRB detected in this study and their experimentally proved dehalogenating activities identified in previous studies

Organism	Organohalides as electron acceptors	Other electron acceptors	References
*Dehalococcoides*	PCE, TCE, DCEs, VC, chlorophenols, chlorobenzenes, polychlorinated biphenyls, -brominated diphenyl ethers	-	[43]
*Dehalogenimonas*	1,2-Dichloroethane, 1,2-dichloropropane, 1,1,2-Trichloroethane	-	[43]
*Desulfitobacterium*	PCE, TCE, 3-chloro-4-hydroxyphenylacetic acid, 1,2-Dichloroethane, Dichlorophenol and tri-chlorophenol	Sulphate	[43, 45]
*Desulfomonile*	3-chlorobenzoate	Sulfite, sulphate and thiosulphate,	[43]
*Desulfovibrio*	2-chlorophenol and 2,6-dichlorophenol	Sulphate	[43]
*Anaeromyxobacter*	2-chlorophenol, 2,6-dichlorophenol and 2,5-dichlorophenol	Nitrate and fumarate	[43, 46, 47]
*Desulfuromonas*	PCE and TCE	fumarate, polysulfide and Fe(III) nitriloacetate	[43, 48]
*Shewanella*	PCE	Fe(III), Mn(IV) and U(VI)	[43, 49]
*Geobacter*	PCE, TCE	Fe(III) and Mn(IV)	[43, 50]

## Discussion

Despite their established role as crucial microbial sinks [10, 11], the potential of mangrove sediments for bioremediation and removal of toxic pollutants like organohalides remains largely unexplored. Previous studies, such as Ding and He [51], suggest that the intrinsic bioremediation capacity of sediments is linked to the activity and distribution of indigenous bacteria, particularly those possessing reductive dehalogenation capabilities. This study investigates the microbial communities associated with five mangrove sediments, focusing on genera identified as OHRB, key players in the efficient and effective bioremediation of organohalides under anaerobic conditions. Additionally, an enrichment culture experiment assesses the actual ability of these mangrove sediments to bioremediate organohalides using PCE as a representative example.

After excluding unclassified sequences from our samples, *Proteobacteria* emerged as the dominant phylum in four examined microbiomes, aligning with established trends in mangrove sediments [52–54]. Notably, *Bacteroidetes* ranked second in two samples, mirroring findings from the Beibu Gulf and Red Sea [55, 56]. However, our results diverged from Beilun Estuary, where *Chloroflexi* held the second-most abundant position [56], and the Bay of Bengal, where *Cyanobacteria* and *Acidobacteria* dominated [57]. These variations highlight the influence of regional and environmental factors on mangrove sediment microbiome composition.

This study identified diverse OHRB and PCE dechlorination activity in mangrove sediments along Al Rayyis White Head (Red Sea, KSA). Notably, obligate OHRB belonging to the genera *Dehalococcoides* and *Dehalogenimonas* were detected in two sediment samples, both affiliated with the phylum *Chloroflexi*. Interestingly, phylum *Chloroflexi* was absent in sediment MS3, where *Proteobacteria* was not the dominant phylum. The presence of obligate OHRB like *Dehalococcoides* and *Dehalogenimonas* strongly suggests potential OHR activity in these sediments, as this dehalorespiration process serves as their sole energy and growth source [58–60]. These findings highlight the potential role of specific bacterial taxa in mediating organohalide biodegradation in these mangrove ecosystems.

Several studies support the role of *Dehalococcoides* (phylum *Chloroflexi*) in mangrove sediment biodegradation. For instance, Dehalogenating *Dehalococcoides* sp. has been previously reported for the anaerobic removal of BDE-47 [61]. Additionally, Liang et al. [62] described interactions between obligate OHRB like *Dehalococcoides* and non-obligate OHRB like *Geobacter* (phylum *Proteobacteria*) during OHR. These interactions might include syntrophic cooperation or synergistic growth, potentially explaining the high relative abundance of *Proteobacteria* observed in our study and its possible positive correlation with *Chloroflexi* abundance, OHRB presence, and OHR activity.

Beyond *Dehalococcoides*, our study identified various non-obligate OHRB genera, including *Anaeromyxobacter*, *Desulfuromonas*, *Geobacter*, *Desulfomonile*, *Desulfovibrio*, *Shewanella* (phylum *Proteobacteria*) and *Desulfitobacterium* (phylum *Firmicutes*). Notably, *Desulfitobacterium* encompasses numerous strains capable of dechlorinating both aliphatic and aromatic hydrocarbons [45], suggesting its potential contribution to biodegradation processes in these sediments.

PICRUSt analysis identified functional biomarkers for OHR, including PCE and Cl-OHPA reductive dehalogenases, confirming the potential for OHR activity within our sediment samples. To our knowledge, this is the first report of PICRUSt-predicted reductive dehalogenases in mangrove sediments. Notably, PICRUSt also revealed functional biomarkers for diverse anaerobic metabolic activities, exhibiting higher relative abundances than aerobic pathways. These include DNRA, anaerobic hydrocarbon biodegradation, and methanogenesis. This suggests a specialization of the mangrove sediment microbiome towards anaerobic processes, potentially supporting ecosystem productivity through DNRA-mediated nitrogen cycling [63, 64]. Furthermore, previous studies have demonstrated the role of mangrove sediments in anaerobic hydrocarbon biodegradation of polycyclic aromatic hydrocarbons [65, 66] and petroleum oil [67], while methanogenesis has been established as a significant contributor to global methane emissions, with mangrove wetlands playing a major role [68–70]. Anaerobic bacterial capabilities were recoded before at a depth of 10–15 cm for aromatic hydrocarbon degradation using a mangrove sediment [66] and at a depth of 11–15 cm for methanogenesis using a lake sediment [71].

GC-FID analysis of PCE-spiked enrichment culture revealed DCE as the primary daughter product, followed by minor VC and ethene detections. This pattern suggests potential involvement of various OHRB in PCE dechlorination to DCE, while the subsequent transformation to non-toxic ethene might be driven by *Dehalococcoides*, as reported previously [72]. This aligns with Maymo-Gatell et al.‘s observation of slower *Dehalococcoides* growth on DCE compared to other electron acceptors [73], although the reason for this remains unclear.

Previous studies have utilized molecular tools to detect OHRB in mangrove sediments. Pan et al. [61] employed qRT-PCR to confirm the presence of OHRB, including *Dehalococcoides*, and suggested their potential contribution to PBDE bioremediation. While T-RFLP and clone library analyses revealed PBDE degradation in mangrove sediments, no significant correlation between *Dehalococcoides* abundance and debromination activity was observed [74]. Additionally, Chen et al. [75] identified OHRB genera (*Dehalobacter*, *Dehalococcoides*, *Dehalogenimonas*, and *Desulfitobacterium*) in a BDE-47 degrading microcosm enriched with mangrove sediment and biochar. However, unlike our study, none of these investigations employed environmentally benign PCE as the organohalide source or implemented PICRUSt analysis to specifically confirm the presence of functional OHR biomarkers (reductive dehalogenases).

## Conclusion

This study provides compelling evidence for the intrinsic bioremediation and dechlorination potential of PCE within mangrove sediments collected from Al Rayyis White Head (Red Sea, KSA). Anaerobic enrichment cultures experiments directly demonstrate this capability, while 16S rRNA metagenomic analysis reveals the presence of diverse OHRB potentially involved in the process. Importantly, the study provides strong evidence for the existence of both OHRB and their activity through the identification of key functional biomarkers, reductive dehalogenases using the PICRUSt tool. These findings significantly contribute to our understanding of how indigenous microbes in mangrove sediments mediate PCE dechlorination and bioremediation. Moreover, they highlight the exciting possibility of enriching and isolating specific microbes from these sediments for further bioremediation applications.

## Data Availability

The raw amplicon sequencing datasets obtained in this study were deposited into National Center for Biotechnology Information (NCBI) Sequence Read Archive (SRA) database and are available under accession number PRJNA987345.
