# Correction: Metagenomic 16S rRNA analysis and predictive functional profiling revealed intrinsic organohalides respiration and bioremediation potential in mangrove sediment

**DOI:** 10.1186/s12866-024-03360-y

**Published:** 2024-06-08

**Authors:** Sultan M. Alsharif, Mohamed Ismaeil, Ali M. Saeed, Wael S. El-Sayed

**Affiliations:** 1 Department of Biology, College of Science, Taibah University, Al-Madinah, Kingdom of Saudi Arabia; 2 Microbiology Department, Faculty of Science, Ain Shams University, Cairo, Egypt

**Correction:** ***BMC Microbiol*** **24, 176 (2024)**

10.1186/s12866-024-03291-8

Following publication of the original article [1], the authors want to change the sequence of the author names in the author list. The sequence of the affiliations should also be changed accordingly. The original article has been corrected.

The sequence of the author names currently reads:

Mohamed Ismaeil^1*^, Sultan M. Alsharif^2^, Ali M. Saeed^1^ and Wael S. El Sayed^1^.

The sequence of the affiliations currently reads:

^1^ Microbiology Department, Faculty of Science, Ain Shams University, Cairo, Egypt.

^2^ Department of Biology, College of Science, Taibah University, Al Madinah, Kingdom of Saudi Arabia.

The sequence of the author names should read:

Sultan M. Alsharif^1^, Mohamed Ismaeil^2*^, Ali M. Saeed^2^ and Wael S. El Sayed^2^.

The sequence of the affiliations should read:

^1^ Department of Biology, College of Science, Taibah University, Al Madinah, Kingdom of Saudi Arabia.

^2^ Microbiology Department, Faculty of Science, Ain Shams University, Cairo, Egypt.
